# Clonal Distribution and Virulence of *Campylobacter jejuni* Isolates in Blood

**DOI:** 10.3201/eid1910.121537

**Published:** 2013-10

**Authors:** Benjamin Feodoroff, Caroline P.A. de Haan, Patrik Ellström, Seppo Sarna, Marja-Liisa Hänninen, Hilpi Rautelin

**Affiliations:** University of Helsinki, Helsinki, Finland (B. Feodoroff, C.P.A. de Haan, S. Sarna, M.-L. Hänninen);; University of Uppsala, Uppsala, Sweden (P. Ellström, H. Rautelin)

**Keywords:** bacteremia, Campylobacter jejuni, bacteria, infections, multilocus sequence typing, sequence type, clonal distribution, virulence, blood, isolates, ST-677, invasive pathogen, Finland

## Abstract

*Campylobacter jejuni* bacteria are highly diverse enteropathogens. Seventy-three *C. jejuni* isolates from blood collected in Finland were analyzed by multilocus sequence typing and serum resistance. Approximately half of the isolates belonged to the otherwise uncommon sequence type 677 clonal complex. Isolates of this clonal complex were more resistant than other isolates to human serum.

The most common bacterial enteropathogen in industrialized countries is *Campylobacter jejuni*. This bacterium typically causes watery diarrhea with fever and abdominal pain (*1*,*2*). Complications, such as bacteremia, Guillain-Barré syndrome, and reactive arthritis, might also occur (*3*).

Multilocus sequence typing (MLST) has shown that *C. jejuni* is weakly clonal and highly diverse (*4*,*5*). Several MLST studies have identified particular niches for certain genetically related MLST lineages (*6*,*7*). Thus, MLST is robust in population genetics and source attribution studies.

Susceptibility to human serum varies between different species of *Campylobacter*; *C. fetus* is typically resistant, and *C. jejuni* is believed to be sensitive (*8*). Because serum resistance might contribute to spread of *C. jejuni* in the bloodstream, systemic isolates have been studied for their survival in human serum. However, numbers of isolates studied have been limited, and results compared with those for fecal isolates have not been distinctive (*8*,*9*).

In a recent nationwide study over a 10-year period, we collected blood culture isolates of *C. jejuni* and *C. coli* and obtained clinical features of corresponding bacteremic episodes and characteristics of patients throughout Finland (*10*). Our results showed that patients were moderately young and mostly without any underlying diseases (*10*). In the present study, we characterized *C. jejuni* blood culture isolates with respect to their clonal distribution and serum resistance.

## The Study

The bacterial isolates were collected throughout Finland during 1998–2007 as described (*10*). Of 76 patients described, 3 were excluded because of *C. coli* infections. MLST was performed for 73 *C. jejuni* isolates as described (*11*). BioNumerics version 5.1 software (Applied Maths, Kortrijk, Belgium) was used for sequence assembly. Allele numbers, sequence types (STs), and clonal complexes (CCs) were assigned by using the PubMLST database (*5*). New alleles and STs were submitted to the database.

A serum sensitivity assay was conducted with 73 *C. jejuni* isolates according to a described protocol (*8*). The same pool of serum samples from 10 healthy blood donors was used in all experiments. *C. jejuni* NCTC 11168 and a *C. fetus* isolate from blood were used as control organisms.

All statistical analyses were performed by using Graphpad Prism version 4.03 (Graphpad Software, San Diego, CA, USA) and PASW Statistics version 18 (SPSS Inc., Chicago, IL, USA). The χ^2^ test and Fisher exact test were used for comparison of categorical variables. The Mann-Whitney test was used for the comparison of continuous variables. All tests were 2-sided, and p<0.05 was considered significant.

A total of 72 *C. jejuni* isolates from blood were successfully typed by MLST; 1 isolate had a mixed MLST pattern. Five isolates were in unassigned STs, and the rest were distributed among 11 CCs (Table). ST-677 CC was the predominant complex: 35 (48%) isolates. Genetic relatedness of these isolates was further confirmed by using pulsed-field gel electrophoresis.

**Table:** Distribution of MLST clonal complexes and sequence types among 73 blood culture isolates of Campylobacter jejuni*

Clonal complex	No. (%)	ST	No.	Characteristics significantly (p<0.05) associated with clonal complex
ST-677	35 (48)	677	27	Serum resistance
		794	8	
ST-45	12 (16)	11	4	Serum sensitivity
		45	3	
		137	2	
		230	2	
		5201	1	
ST-21	10 (14)	50	5	Underlying disease and longer duration of hospitalization of patients
		883	2	
		1948	1	
		5670	1	
		Uncertain	1	
ST-48	2 (3)	38	1	ND
		48	1	
ST-464	2 (3)	464	1	ND
		3140	1	ND
ST-52	1 (1)	52	1	ND
ST-354	1 (1)	3155	1	ND
ST-443	1 (1)	5671	1	ND
ST-460	1 (1)	606	1	ND
ST-508	1 (1)	508	1	ND
ST-1332	1 (1)	1332	1	ND
Unassigned	5 (7)	468	1	ND
		1080	1	ND
		1972	1	ND
		5673	1	ND
		5674	1	ND
Mixed	1 (1)	Uncertain	1	ND

*MLST, multilocus sequence typing; ST, sequence type; ND, none detected.

**Figure 1:**
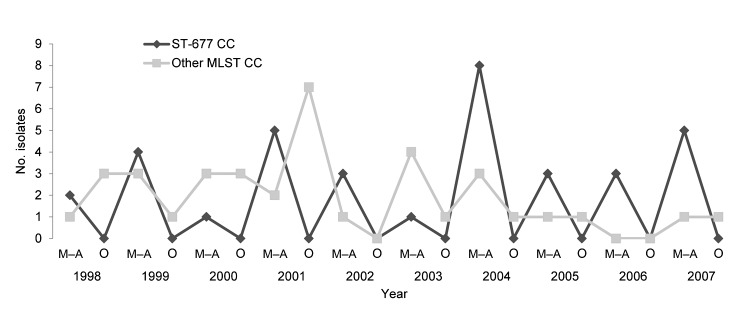
Annual and seasonal distribution of 72 *Camplyobacter jejuni* blood culture isolates belonging either to the ST-677 clonal complex (CC) or to the other multilocus sequence typing (MLST) CCs. One isolate with a mixed multilocus sequence type was not included. *C. jejuni* bacteremia was diagnosed during May–August (M–A) or during any other month of the year (O).

Isolates belonging to ST-677 CC were obtained throughout the 10-year study period. However, bacteremia episodes caused by ST-677 CC isolates were exclusively diagnosed during the seasonal peak during May–August (Figure 1). Of *C. jejuni* blood culture isolates detected during May–August, most (64%) were ST-677 CC. Furthermore, ST-677 CC was the most prevalent complex in 4 geographic regions of Finland.

**Figure 2:**
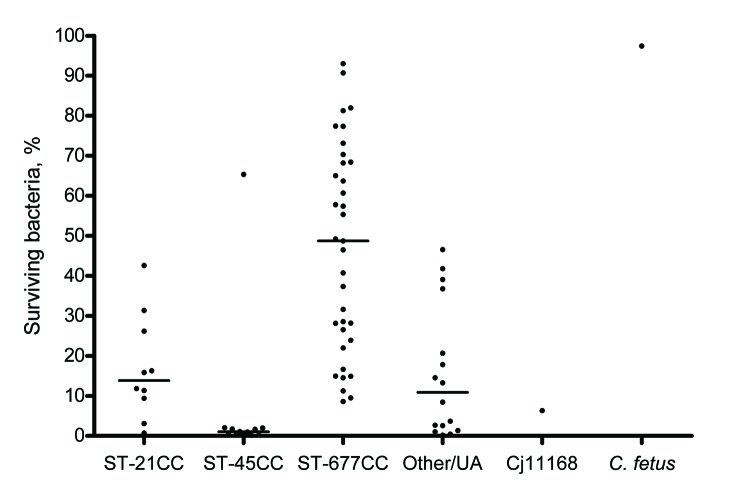
Percentage of surviving bacteria in human serum for 73 blood culture isolates of *Campylobacter jejuni* (Cj), grouped according to major multilocus sequence typing clonal complexes (CCs), and for controls *C. jejuni* Cj11168 and *C. fetus*. Dots indicate mean values for 2–3 experiments. Horizontal lines indicate median values for each CC group. ST, sequence type; UA, unassigned.

Susceptibility to human serum varied between *C. jejuni* isolates from different CCs (Figure 2). ST-677 CC isolates were significantly less susceptible to human serum than all other isolates (p<0.0001). ST-45 CC isolates were significantly more susceptible to human serum than all other isolates (p<0.0001).

## Conclusions

We characterized a unique collection of 73 *C. jejuni* isolates from blood obtained during a nationwide study in Finland over a 10-year period. Despite the high population diversity of *C. jejuni*, nearly half of the isolates from blood showed clustering within the ST-677 CC, a rare CC in other countries (*12*,*13*). Furthermore, bacterial survival in human serum varied greatly. Thus, invasiveness of blood culture isolates could not be solely explained by their serum resistance, although the predominant isolates of ST-677 CC were more serum resistant than other isolates.

*C. jejuni* has high ST diversity. As of May 2, 2013, a total of 6,564 STs were registered (*5*). In this study, we detected clustering of *C. jejuni* isolates from blood in an uncommon ST-677 CC. Further studies are needed to clarify whether bacterial characteristics might explain this finding.

In our previous study, which included human fecal *C. jejuni* isolates obtained in Finland from the mid-1990s through 2007, which is nearly the same period as in the current nationwide study, 11.7% of the isolates belonged to ST-677 CC (*11*). The 2 most prevalent CCs in that study, ST-45 CC (43.6% of fecal isolates) and ST-21 CC (19.4% of fecal isolates), were detected only among 12 (16%) and 10 (14%) of blood culture isolates, respectively, in the present study.

ST-45 CC and ST-21 CC have been shown to be prevalent in several countries (*4*,*13*). However, our results suggest that these 2 CCs are not common among *C*. *jejuni* isolates from blood in Finland, which cluster more in the ST-677 CC. On the basis of the present results, we speculate that ST-677 CC might have a special invasive capability or has adapted to the environment in Finland.

In general, complement-mediated killing of serum-susceptible isolates plays a major role in restricting access of pathogens to the bloodstream. However, available information about possible serum sensitivity of *C. jejuni* isolates from blood is scarce (*8*,*9*). In our study of nonselected *C. jejuni* isolates from blood, susceptibility to human serum varied according to MLST CC.

In conclusion, in this nationwide study during a 10-year period in Finland, we found by MLST analysis that half of the bacteremia isolates of *C. jejuni* clustered within an otherwise uncommon ST-677 CC. Whether this finding indicates special adaptation of ST-677 CC to Finland or to the human bloodstream is not clear and needs to be studied. Our findings emphasize the role of using well-defined clinical materials in studies on bacterial pathogenicity and severity of human disease.
